# Cumulative Risk on Oxytocin-Pathway Genes Impairs Default Mode Network Connectivity in Trauma-Exposed Youth

**DOI:** 10.3389/fendo.2020.00335

**Published:** 2020-05-22

**Authors:** Maor Zeev-Wolf, Jonathan Levy, Richard P. Ebstein, Ruth Feldman

**Affiliations:** ^1^Department of Education, Zlotowski Center for Neuroscience, Ben Gurion University of the Negev, Beer Sheva, Israel; ^2^Interdiscilinary Center Herzliya, Baruch Ivcher School of Psychology, Herzliya, Israel; ^3^Department of Neuroscience and Biomedical Engineering, Aalto University, Espoo, Finland; ^4^Department of Psychology, National University of Singapore, Singapore, Singapore; ^5^Child Study Center, Yale University, New Haven, CT, United States

**Keywords:** OXTR, magnetoencephalography, trauma exposure, anxiety disorders, longitudinal studies, genetics

## Abstract

**Background:** Although the default mode network (DMN) is a core network essential for brain functioning, little is known about its developmental trajectory, particularly on factors associated with its coherence into a functional network. In light of adult studies indicating DMN's susceptibility to stress-related conditions, we examined links between variability on oxytocin-pathway genes and DMN connectivity in youth exposed to chronic war-related trauma

**Methods:** Following a cohort of war-exposed children from early childhood, we imaged the brains of 74 preadolescents (age 11–13 years; 39 war-exposed) during rest using magnetoencephalography (MEG). A cumulative risk index on oxytocin-pathway genes was constructed by combining single nucleotide polymorphisms on five genes previously linked with social deficits and psychopathology; *OXTR* rs1042778, *OXTR* rs2254298, *OXTR*rs53576, *CD38* rs3796863, and *AVPR1A* RS3. Avoidant response to trauma reminders in early childhood and anxiety disorders in late childhood were assessed as predictors of disruptions to DMN theta connectivity.

**Results:** Higher vulnerability on oxytocin-pathway genes predicted greater disruptions to DMN theta connectivity. Avoidant symptoms in early childhood and generalized anxiety disorder in later childhood were related to impaired DMN connectivity. In combination, stress exposure, oxytocin-pathway genes, and stress-related symptoms explained 24.6% of the variance in DMN connectivity, highlighting the significant effect of stress on the maturing brain.

**Conclusions:** Findings are the first to link the oxytocin system and maturation of the DMN, a core system sustaining autobiographical memories, alteration of intrinsic and extrinsic attention, mentalization, and sense of self. Results suggest that oxytocin may buffer the effects of chronic early stress on the DMN, particularly theta rhythms that typify the developing brain.

## Introduction

The default mode network (DMN) is a cortical network that becomes more active and synchronized during rest and deactivates when one is engaged in a goal-oriented task (1). The DMN comprises seven brain regions, including the left and right angular gyrus, ventro- and dorso-medial pre-frontal cortex, posterior cingulate cortex/pre-cuneus, right medial pre-frontal cortex, and left inferior temporal gyrus (2), and its organization and functioning during rest is considered to define the personal “fingerprint” of the brain (3). In adults, the DMN is thought to play a key role in sustaining the sense of self and in organizing the individual's experience of the world; it supports self-referential thinking, introspection (4), internal narratives (4), mentalizing activity (5), the autobiographical self and self-projection (6). Highly synchronized and smooth activity of the DMN is critical for a sense of well-being, personal identity, and resilience (7–11).

The importance of the DMN for proper brain functioning is widely-accepted. The DMN is involved in overall brain activity as well as in attentive, cognitive, and social processes, and associations have been repeatedly shown between DMN dysfunction and a host of psychiatric disorders in adults (12–16). In contrast, very little is currently known about the development and functional connectivity of the DMN in children and particularly lacking are data related to DMN maturation under high-risk conditions, with even more limited knowledge on how structures of the DMN cohere in children reared under various high-risk conditions. A study utilizing resting state functional connectivity MRI and graph theory methods to characterize the maturation of the DMN found that in contrast to the strong interhemispheric connectivity between homolog brain regions in adult, DMN connectivity in children is sparse and becomes more integrated with development (17). The authors interpret their findings in relation to the ever-maturing functions sustained by the DMN, such as consolidation of the sense of self that is still immature in children. For example, the ability to encode and retrieve stored memories is fully functioning at an early age but episodic memory continues to improve with age. This improvement does not stem from the mere development of encoding and retrieval but from the ability to incorporate more complex strategies to encode self-relevant information, which is supported by the progressive integration of the DMN into a unified network. These notions are supported by a study indicating that DMN connectivity in children and adolescents is related to the quality of reconstruction of past experiences and the ability to imagine the future (18).

Very few studies tested the maturation of DMN connectivity across childhood and adolescence. A longitudinal fMRI study found significant changes in DMN connectivity between the ages 10 and 13, suggesting that it continues to consolidate throughout development (19). This is consistent with cross-sectional studies that compared DMN connectivity in children, adolescents, and adults (17, 20–23). Notably, the findings that age-related differences are observed even within the narrow age-range of 10–13 lends further support to the hypothesis that early adolescence may be a particularly important time-window for the integration of the DMN into a coherent network.

While very little research has tested DMN connectivity using magnetoencephalography (MEG), MEG has unique benefits for the study of DMN functionality due to its high temporal resolution which enables the decomposing of neural signals into frequency bands (24). Using MEG/EEG studies it was found that whereas in adults, the strongest activity in the DMN during rest is in the alpha band (8–12 Hz), during childhood the dominant frequency is the theta band (4–7 Hz), which gradually shifts to alpha as children grow (25–28). In a previous longitudinal study we utilized MEG to investigate the effect of early life stress (ELS) on DMN connectivity in preadolescents and their mothers and found that ELS decreased theta DMN connectivity in adolescents and alpha DMN connectivity in mothers (29); hence, we focused here on theta connectivity in youth by assessing Phase Locking Value [PLV; Lachaux et al. (30)], the most commonly used index to measure phase coupling in EEG/MEG experiments (31, 32). PLV quantifies the degree to which signals rise and fall together over time in absolute values; high PLV (values closer to 1) implies more synchronized signals while low PLV (values closer to 0) denotes lower synchrony. We found that the experience of anxious, intrusive parenting across the first decade and higher cortisol levels predicted DMN connectivity impairment, indicating that neurobiological, clinical, and relational factors that index stress longitudinally shape DMN connectivity in children.

The neuropeptide oxytocin (OT) has been repeatedly implicated in attachment, bonding, and sociality across mammalian species (33, 34). In addition to its involvement in bond formation, OT plays a critical role in stress regulation. In response to emotional, physical, or pharmacological stress, OT is released into the bloodstream as well as within hypothalamic and extrahypothalamic limbic regions where it regulates HPA activity and modulates response to stress (35–37). Since the DMN is highly sensitive to stress, its functionality is probably impacted by the OT system and evidence points to the association between OT and DMN activity in adults (38–40). One study describes associations between allelic variability on the oxytocin receptor gene (*OXTR*) and DMN connectivity (40). Following research indicating that 40% of the variance in DMN connectivity is heritable, Wang et al. (40) found that compared with individuals with *OXTR*rs2254298 GG genotype, individuals carrying the AA/AG genotypes displayed lower DMN connectivity.

To further understand the association between genetic variability on the *OXTR* and stress-related conditions, studies have suggested that a cumulative genetic risk index—a measure combining multiple single nucleotide polymorphisms (SNPs) on oxytocin-pathway genes linked with social dysfunction—provides a better risk estimate than the study of individual SNPs (41–44). Three *OXTR* SNPs have been typically included in this cumulative index, the rs2254298, rs53576, and rs1042778 alleles. In addition to these three alleles, *CD38*, a multifactorial molecule implicated in cell proliferation, differentiation, and migration that plays a critical role in regulating OT release (45) and vasopressin *AVPR1a*, a closely linked neuropeptide to OT that is considered to evolve from the same ancient vasotocin molecule (46), have been incorporated into the oxytocin-pathway genes cumulative index. This cumulative risk index, combining the three *OXTR* SNPs, *CD38*, and *AVPR1a*, has been shown to predict PTSD chronicity in children exposed to chronic trauma (47) and to correlate with empathic failure among new romantic partners (48). Furthermore, the cumulative risk approach is more statistically powerful and enables the evaluation of a continuum of genetic risk in relation to stress-related conditions.

**Figure 1:**
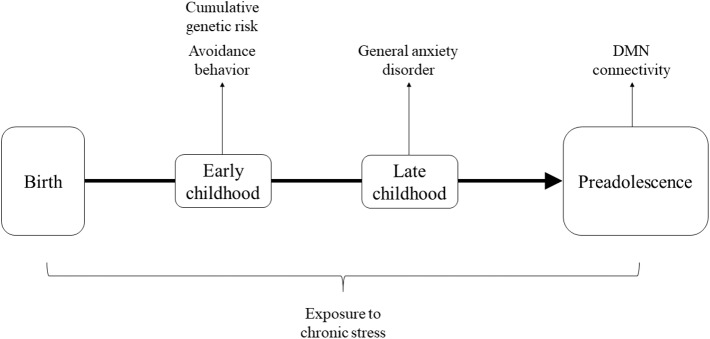
Theoretical framework describing factors across development hypothesized to cumulatively impact maturation of DMN connectivity.

In light of the above, the current study utilized a unique cohort of stress-exposed children living in Sderot, Israel, a small town located on the Gaza boarder which has been subjected to continuous, life-threatening trauma for nearly two decades. Sderot has been the target of continuous rocket attacks since 2001, with six periods of exacerbations when the town was under attack by dozens of daily missiles for several days to several weeks. During attacks, citizens hear a siren warning, leaving them only 15 s to reach shelters before an explosion. Children were followed at several time-points from early childhood and at the transition to adolescence (11–13 years) underwent MEG scanning to assess DMN connectivity. Our goal was to examine the cumulative contributions of genetic susceptibility on the OT system and anxiety-related symptoms, which are known to jointly impact neural responses (49, 50), to the development of DMN connectivity in children growing in the context of chronic trauma. We thus examined stress-related factors across childhood and hypothesized that cumulatively these factors may place children at risk for maturation of the DMN. Specially, we considered genetic variability on the OT system, avoidant manifestations in early childhood, and the consolidation of a full-blown anxiety disorder in late childhood as factors which, when occurring together across development, may tip children toward a risk trajectory. Our conceptual model is presented in Figure 1.

In early childhood, we focused on children's avoidant responses to trauma reminders. Avoidance is a core symptom cluster in the diagnosis of PTSD and has shown to predict PTSD chronicity in children and adults (47, 51). The diagnosis of PTSD in early childhood, before children can verbally recount the trauma, requires clinical observation of the child's behavioral response to trauma reminders [DC: 0–3R; Zero to Three (52)]. Young children's behavioral response to trauma reminders is characterized by four types of responses; regressive-clinging, aggressive, hyper-anxious, and avoidant (53), of which the avoidant type is the most detrimental for later development (47). In terms of anxiety disorders, longitudinal studies has shown that temperamental avoidance and reticence in early childhood increase the risk for the consolidation of anxiety disorders by late childhood and adolescence, and only those children who showed both reticence in early childhood and anxiety disorders later on exhibited alterations in neural functioning (54–57). We thus examined the joint contribution of early behavioral avoidance and later anxiety disorders to DMN connectivity at the transition to adolescence.

Several hypotheses were formulated. First, we expected that greater cumulative risk index on oxytocin-pathway genes would be associated with reduced connectivity of the DMN (hypothesis 1). We also expected that avoidant response to trauma reminders in early childhood would be linked with impaired DMN connectivity (hypothesis 2). Similarly, the existence of a full-blown anxiety disorder, which increases the likelihood of a chronic course of PTSD (51), was expected to predict lower DMN connectivity (hypothesis 3). Finally, these factors in combination—genetic risk, avoidant response, and anxiety disorder, were expected to jointly explain variance in DMN connectivity in preadolescence (hypothesis 4).

## Methods

### Participants

The original sample included 232 children in two groups (war-exposed and control) who were observed four times. In the current study we used data collected in early childhood (T1), late childhood (T3), and early preadolescence (T4). Details of the first (T1) second assessment (T2) appear elsewhere (53, 58).

#### T1: Early Childhood

In early childhood (time 1 [T1]), the initial sample (*n* = 232) included 47.6% boys and 47.1% firstborns (*M* = 2.76 years, *SD* = 0.91). The war-exposed group included 148 families living in the same neighborhoods in Sderot, Israel, located 10 km from the Gaza border. The control group included 84 non-exposed families from comparable towns in the Tel-Aviv area matched to the exposed group by age, gender, birth order, parental age and education, maternal employment, and marital status and subsequently screened for trauma.

#### T3: Late Childhood

At late childhood (*M* = 9.3 years, *SD* = 1.41), 177 families were revisited: 101 war-exposed and 76 control families. Attrition was mainly related to inability to locate families or families relocating to another city.

#### T4: Pre-adolescence

A total of 166 families, including 111 children, were revisited at preadolescence. Out of the 111 children revisited, 74 children (43 girls), containing 39 war-exposed children, participated in MEG scanning (*M* = 11.81 years, *SD* = 1.24). Table 1 presents demographic and background information. Of 111 children initially recruited at T4, 36 did not yield MEG data: 18 were MEG incompatible (mostly owing to metal implants), 6 refused MEG scan, and 12 did not complete the experiment.

**Table 1:** Demographic details.

	**War-exposed**		**Control**		***t***	***p***
	**Mean**	**SD**	**Mean**	**SD**		
Age	11.93	1.35	11.59	1.12	1.15	n.s.
Rooms	4.26	1.06	4.37	1.28	−0.41	n.s.
IQ-P T3	11.16	2.56	11.34	2.52	−0.29	n.s.
IQ-V T3	11.49	2.64	11.13	2.76	0.56	n.s.
	**Boys**	**Girls**	**Boys**	**Girls**	**χ^2^**	***p***
Gender	22	17	10	26	4.5	0.034
	**Firstborn**	**Not first**	**Firstborn**	**Not first**		
Birth order	16	23	21	14	2.65	n.s.

*Rooms, Number of rooms in the house; IQ-P, Standardized scores from blocks subtest from the WISC-R-95 as measured at T3; IQ-V, Standardized scores from vocabulary subtest from the WISC-R-95 as measured at T3*.

The study was approved by local institutional review board, and written informed consent was obtained from parents after receiving a complete description of the study.

### Procedure and Measures

#### T1: Early Childhood

During the home visits (which lasted 3.5 h on average), DNA samples were collected from the children. In addition, trained clinicians interviewed mothers about their children and children were observed during trauma reminders, to diagnose PTSD [detailed in Feldman and Vengrober (53)]. Home visits included other procedures which exceed the scope of the current study and which are described elsewhere (53, 59).

##### DNA collection

DNA of child was extracted from 20 ml of mouthwash samples using Master Pure kit (Epicenter, Madison WI). All genotype frequencies of *OXTR, CD38*, and *AVPR1A* SNPs were in Hardy-Weinberg equilibrium [for full details on genotyping see references (48, 60–65)]. A cumulative genetic risk factor was computed for each child by summing the number of genetic risk variations associated with psychiatric risk or social difficulties (Table 2). These included the *OXTR* rs1042778 TT genotype, associated with greater risk for autism (65) and lower empathy in healthy adults (66); *OXTR* rs2254298 GG genotype, associated with smaller amygdala volume in two independent samples (67, 68) and risk for major depression (60); the A allele (AA or AG) on the *OXTR*rs53576, related to risk for autism (69), lower empathy (70), and non-optimal parenting (62); the CC genotype on the *CD38* rs3796863, linked with greater risk for autism in two independent samples (71, 72) and non-optimal parenting (63, 64); and the 327 bp allele on the *AVPR1A* RS3, associated with lower altruism and non-optimal mothering (61, 73, 74). Risk scores ranged from 0 (no risk) to 5 (risk on all 5 SNPs). In total DNA was extracted from 231 children which include the final sample of 74 children conducting the MEG scan.

**Table 2:** Genotypes on the OXTR, CD38, and AVPR1a genes in the genetic risk index.

**Name**	**Genotype**		**Prevalence of High risk**
	**High risk**	**Low risk**	
OXTR rs1042778	TT	GG, GT	11.5%
OXTR rs2254298	GG	AA, AG	42.6%
OXTRrs53576	AA, AG	GG	44.3%
CD38 rs3796863	CC	AA, AC	33.6%
AVPR1A RS3	327 bp^a^	non-327	22.6%
Cumulative Risk Mean (SD)			1.56 (1.18)

*Cumulative genetic risk is computed for each child by combining the number of high risk genotypes on each of the 5 SNPs*.

a *At least one 327 bp repeat*.

##### Avoidance

During the maternal PTSD interview, which lasted ~1 h, the clinician probed the trauma in detail while children were present in the room and mother and child's behavior during the trauma reminders were videotaped. The child's emotions and behaviors to trauma reminders were coded offline by raters blinded to child psychiatric information along 15 scales, each rated from 1 (low) to 5 (high). Coding of trauma response was based on the Coding Interactive Behavior [CIB, Feldman (75)] and described in detail elsewhere (53). The CIB is a well-validated coding system for adult-child interaction and child behavioral response that has shown good psychometric properties and sensitivity to adult and child interactive behavior related to age, culture, biological and social-emotional risk conditions, and the effects of intervention (76–79). Interrater reliability was conducted for 46 interactions and reliability averaged 93%, intraclass *r* = 0.91. Child avoidance behavior included child gaze aversion, emotional withdrawal, moving away from mother's arms' reach, and increased and inappropriate preoccupation with objects (α = 0.81).

#### T3: Late Childhood

During the 3 h home visit, general anxiety disorder in children was assessed via interviewing mothers. Home visits included other procedures which exceed the scope of the current study and which are described elsewhere (58).

##### Generalized anxiety disorder (GAD)

The developmental and Well-Being Assessment was used to diagnose child Axis-I disorders, including GAD. The DAWBA is a structured interview generating ICD-10 and DSM-IV psychiatric diagnoses in 5–17-years-old children (80). The DAWBA, administered to mothers, is well-validated, including a large epidemiological study in Israel (81). The DAWBA was administered by clinicians and supervised by child psychiatrist, blind to any other information, with reliability > 85% and cases conferred every few weeks. GAD scores ranged between 0 (i.e., lack of GAD symptoms) to 17 (i.e., severe GAD).

#### T4: Pre-adolescence

Children visited with their mothers the MEG unit at Bar Ilan University where they underwent a magnetoencephalography (MEG) scan. MEG scans included recordings of brain activity during rest (data from this stage is used here) as well as an empathy task used elsewhere (82).

##### Default mode network connectivity: phase locking value (PLV)

To avoid discomfort among participants and to minimize artifacts due to restlessness, spontaneous brain activity was measured using MEG while participants rested with open eyes in a dimmed light room for 2 min. Ongoing brain activity was recorded using a whole-head 248-channel magnetometer array (Magnes 3600WH; 4-D Neuroimaging, San Diego, CA) in supine position inside a magnetically shielded room. Data were sampled online at 1017.23 Hz with bandpass of 0.1–400 Hz. Reference coils located ~30 cm above the head oriented by the x-, y-, and z-axes were used to remove environmental noise. Five coils were attached to participants' scalp for recording the head position relative to the 248-sensor array. External noise and heartbeat artifacts were removed using a predesigned algorithm (83). Further data processing and analysis were performed using MATLAB and the FieldTrip toolbox (84). Data were segmented into 1,000 ms epochs (with 500 ms overlap between neighboring epochs). Segments containing muscle artifacts and signal jumps were excluded from further analysis by visual inspection. Data were then filtered in the 1–100-Hz range with 10 s padding (to avoid distortion of the real signal at the ends of segments). To clean eye blinks, eye movements, and leftover heartbeats, spatial component analysis was applied.

DMN seed coordinates were predefined based on prior research (2) in which resting-state MEG data linked with the DMN network defined in functional magnetic resonance imaging studies to detect the equivalent MEG seed coordinates to functional magnetic resonance imaging studies [seed coordinates appear in Zeev-Wolf et al. (29)]. Because children's brain anatomy differs from that of adults, we used different brain templates for children and mothers (Montreal Neurological Institute). The templates were modified to fit each participant's digitized head shape using SPM8 (Wellcome Department of Imaging Neuroscience University College London; http://www.fil.ion.ucl.ac.uk). The head shape was manually digitized (FASTRAK digitizer; Polhemus, Colchester, VT), and the participant's brain volume was divided into a regular grid. Grid positions were obtained by linear transformation in a canonical 1-cm grid. This procedure facilitates group analysis because no spatial interpolation of the volumes of reconstructed activity is required. For each grid position, spatial filters were reconstructed to record activity from location of interest while suppressing other activity.

**Figure 2:**
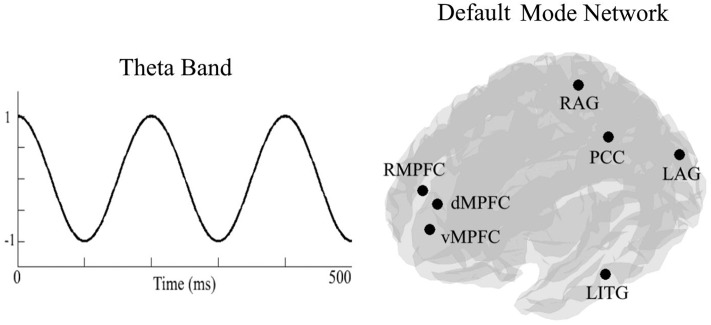
On the left side an, illustration of the theta frequency band (4–7 Hz). On the right side, an illustration of locations of the seven seeds of the default mode network. RMPFC, right medial pre-frontal cortex; dMPFC, dorsomedial pre-frontal cortex; vMPFC, ventromedial pre-frontal cortex; RAG, right angular gyrus; LAG, left angular gyrus; PCC, posterior cingulate cortex/precuneus; LITG, left inferior temporal gyrus.

To calculate DMN theta connectivity, we extracted time series from the activation seeds by applying a linear-constrained minimum-variance beamformer in a predefined frequency band (4–7 Hz). Next, we computed the PLV between all seven seed pairs ($\left(\frac{7}{2}\right)=\;$21 pairs). The PLV measures the phase difference between two recorded signals to quantify the consistency of the phase difference over time (30). See Figure 2 for an illustration of the DMN.

### Statistical Analysis

In order to test the hypotheses stated above, we run three types of analyses: (a) three independent *t*-tests between children with high and low genetic risk, high and low avoidant behavior, and high and low anxiety disorders on DMN connectivity, (b) correlations between all variables to understand the inter-relationships between them, and (c) a regression analysis to test the additive contribution of exposure, genetic risk, avoidant behavior, and anxiety disorder to the variability of DMN connectivity.

## Results

### Genetic Risk on the OXTR and Developmental Outcomes

**Figure 3:**
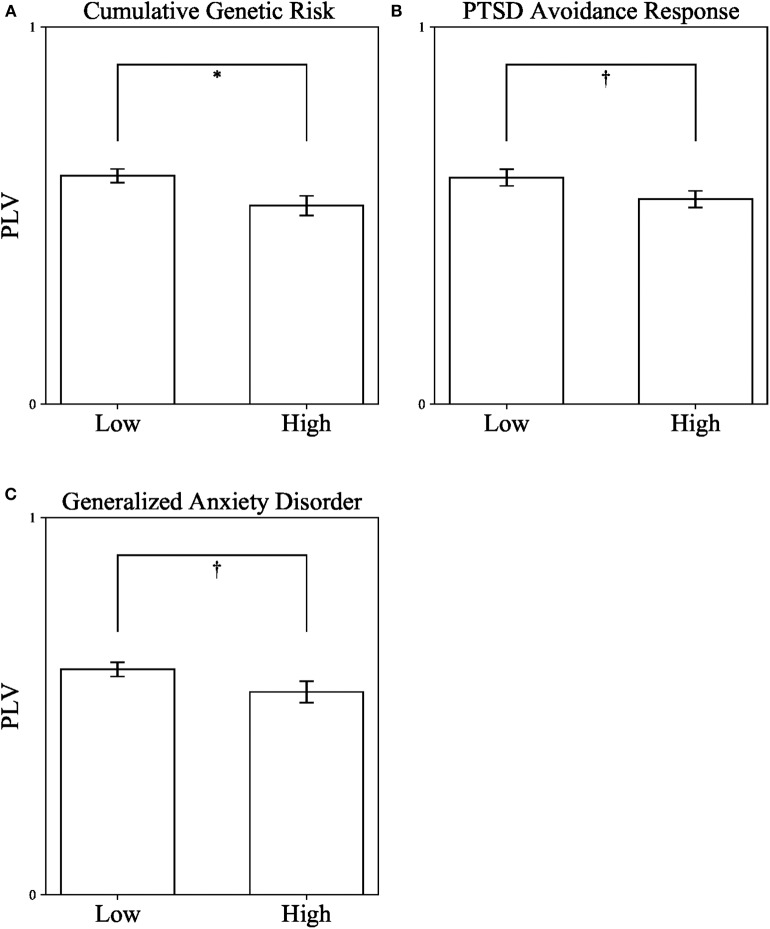
Differences in DMN connectivity (PLV) from a series of independent *t*-tests between children with high and low Cumulative Genetic Risk on Oxytocin-Pathway Genes **(A)**, PTSD Avoidance Response in Early Childhood **(B)**, and Generalized Anxiety Disorder in Late Childhood **(C)**. PLV, Phase locking value. *p < 0.05; †*p* < 0.1. Error bars represent standard errors.

As a first step, we measured DMN connectivity in war-exposed and control children. War-exposed children had lower theta band PLV (*M* = 0.54, *SD* = 0.15) compared to controls (*M* = 0.6, *SD* = 0.11), *t*_(73)_ = 1.99, *p* = 0.05, Cohen's *d* = 0.456, 95% CI [0, 0.121], indicating that exposure to chronic early stress negatively impacts maturation of the DMN. Following, children were divided into high and low groups once, according to the median of cumulative genetic risk from the entire sample (high ≥ 3), once according to the median of avoidance (high ≥ 2), and once according to the median of GAD (high ≥ 7) scores. Three independent *t*-test analyses comparing DMN connectivity (i.e., PLV) in the theta band between children with high (*n* = 32) and low (*n* = 42) cumulative genetic risk, high (*n* = 36) and low (*n* = 38) avoidance, and high (*n* = 42) and low (*n* = 30) GAD scores were conducted. Levene's test indicated that variances were equal between groups in all three analyses. Results show that children with low cumulative genetic risk (*M* = 0.601, *SD* = 0.134) had higher PLV than children with high cumulative genetic risk (*M* = 0.526, *SD* = 0.149), *t*_(73)_ = 2.515, *p* = 0.014, Cohen's *d* = 0.529, 95% CI [0.001, 0.141] (see Figure 3A). A similar trend was found for both avoidance and GAD scores, indicating that children with low avoidance (*M* = 0.601, *SD* = 0.134) had higher PLV than children with high avoidance (*M* = 0.543, *SD* = 0.141), *t*_(72)_ = 1.786, *p* = 0.078, Cohen's *d* = 0.422, 95% CI [−0.006, 0.122] (see Figure 3B); and that children with low GAD scores (*M* = 0.598, *SD* = 0.127) had higher PLV than children with high GAD scores (*M* = 0.538, *SD* = 0.153), *t*_(70)_ = 1.81, *p* = 0.075, Cohen's *d* = 0.427, 95% CI [−0.006, 0.126] (see Figure 3C), though note that the latter two effects did not reach significance. There were no significant interactions between exposure and the other variables: *F*_(1, 73)_ = 0.101, *p* = 0.752 for the interaction with genetic risk; *F*_(1, 73)_ = 1.169, *p* = 0.283 for the interaction with avoidance behavior; *F*_(1, 71)_ = 0.573, *p* = 0.452 for the interaction with GAD.

We *ad-hoc* also looked at differences in cumulative genetic risk, avoidance behavior, and GAD scores between war-exposed and control children. We expected to find differences for GAD scores and avoidance behavior between the groups but not for cumulative genetic risk. Results partly supported our assumptions; avoidance behavior was higher in the exposed children (*M* = 1.653, *SD* = 0.944) compared to controls (*M* = 1.231, *SD* = 0.553), *t*_(198.663)_ = 3.89, *p* < 0.001, Cohen's *d* = 0.525, 95% CI [0.165, 0.679] (equal variances not assumed) and cumulative genetic risk did not differ between the groups [*t*_(229)_ = 1.475, *p* = 0.142]. However, there was also no difference in the prevalence of GAD between groups [*t*_(172)_ = 1.485, *p* = 0.139].

As can be seen in Table 1, there was a gender difference between war-exposed and control children. However, gender was ruled out as an alternative explanation for DMN connectivity since no gender-by-gender or gender-by-exposure effects were found [*F*_(1, 73)_ = 0.001, *p* = 0.99; *F*_(1, 71)_ < 0.001, *p* = 0.99, respectively]. Similarly, while there was a gender difference between children with high and low GAD scores (χ^2^ = 5.634, *p* = 0.018; 8/22 boys/girls in the high GAD group and 19/23 boys/girls in the low GAD group), there was no gender-by-GAD effect [*F*_(1, 72)_ = 2.67, *p* = 1.07]. Furthermore, there were no gender differences between children with high and low genetic risk (χ^2^ = 0.303, *p* = 0.582), high and low avoidance behaviors (χ^2^ = 0.071, *p* = 0.79) or high and low GAD scores (χ^2^ = 0.303, *p* = 0.582).

### Correlation Analysis

**Figure 4:**
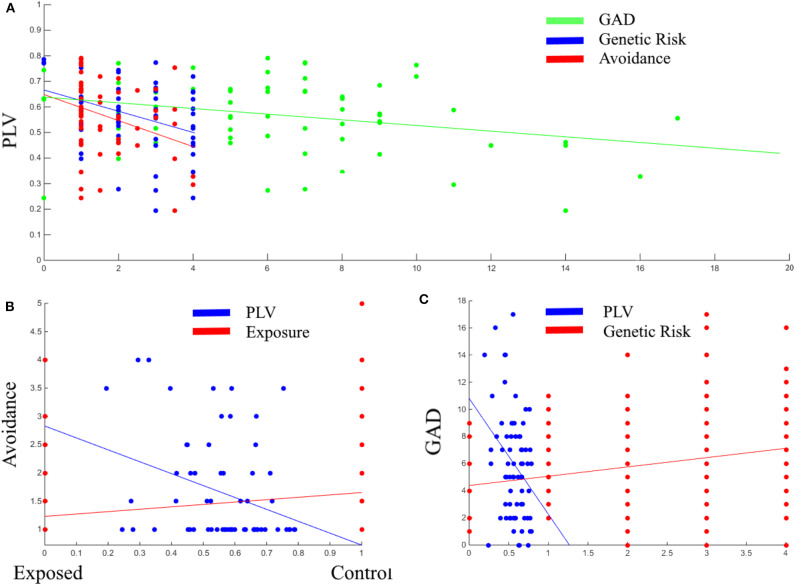
Scatter plots between PLV, GAD, genetic risk, and avoidance **(A)**; Between avoidance, PLV, and exposure **(B)**; Between GAD, PLV, and genetic risk **(C)**. PLV, Phase locking value; GAD, General anxiety disorder scores; Genetic Risk, cumulative genetic risk. Linear lines are regression lines (between the variable on axis Y and the variable in the same color as the regression line).

Next, Pearson correlation analysis was conducted between all five variables across all participants (for full results see Table 3). It was found that DMN connectivity (i.e., PLV) is significantly negatively correlated with cumulative genetic risk (*r* = −0.359, *p* = 0.002), avoidance (*r* = −0.327, *p* = 0.008), and GAD scores (*r* = −0.308, *p* = 0.009), indicating that greater the genetic risk, the more avoidance the child exhibits to trauma reminders and the more severe his/her GAD, the less connectivity the child has in the theta band in the DMN (i.e., lower PLV). Moreover, cumulative genetic risk was significantly positively correlated with GAD scores (*r* = 0.218, *p* = 0.004), indicating that the more genetic risk factors the child has the more severe his/her GAD. In addition, exposure was significantly positively correlated with avoidance (*r* = 0.229, *p* = 0.001), indicating that exposed children are more likely to exhibit avoidance behavior. For scatter plots see Figure 4.

**Table 3:** Correlation coefficients.

	**Exposure**	**Genetic Risk**	**Avoidance**	**GAD**
PLV	−0.158 (0.178)	**−0.359 (0.002)**	**−0.327 (0.008)**	**−0.308 (0.009)**
Exposure		−0.079 (0.233)	**0.229 (0.001)**	0.113 (0.139)
Genetic Risk			−0.021 (0.769)	**0.218 (0.004)**
Avoidance				0.124 (0.127)

*p-values appear in brackets; Genetic Risk, Cumulative Genetic Risk*.

*The bold values indicates significant correlations*.

### Regression Analysis

Lastly, hierarchical regression analysis was conducted to predict DMN theta connectivity (PLV values). Exposure was entered in the first step; cumulative genetic risk was entered in the second step; and avoidance and GAD scores were entered in the third step. This order was chosen to reflect the chronological influence from early to later. Regression statistics are presented in Table 4. The hierarchical regression revealed that at stage one, exposure did not contribute significantly to the regression model, *F*_(1, 60)_ = 1.28, *p* = 0.262, as it accounted only for 2.1% of the variation in PLV. However, introducing cumulative genetic risk explained an additional 13.7% in variation of PLV and this change in *R*^2^ was found significant, *F*_(1, 59)_ = 9.621, *p* = 0.003. Similarly, adding avoidance and GAD scores in the third step explained an additional 8.8% in variation of PLV and this change in *R*^2^ was also found significant, *F*_(1, 57)_ = 3.308, *p* = 0.044. Together, the four variables explained 24.6% of the variance in PLV. As seen in Table 4, the final model indicates that cumulative risk on oxytocin-pathway genes and child avoidant response to trauma reminders in early childhood each uniquely predicted variability in DMN connectivity at the transition to adolescence.

**Table 4:** Summary of hierarchical regression analysis for variables predicting PLV.

**Variable**	**β**	***t***	***SE***	***p***	***R***	***R*^**2**^**	**Δ*R*^**2**^**
Step 1					0.145	0.021	0.021
Exposure	−0.043	−1.131	0.038	0.262			
Step 2					0.398	0.158	0.137
Exposure	−0.044	−1.238	0.035	0.221			
Cumulative genetic risk	−0.44	−3.102	0.014	0.003	95% CI [−0.72, −0.15]		
Step 3					0.496	0.246	0.088
Exposure	−0.012	−0.322	0.036	0.749			
Cumulative genetic risk	−0.038	−2.749	0.014	0.008	95% CI [−0.66, −0.1]		
Avoidance	−0.042	−2.125	0.02	0.038	95% CI [−0.08, −0.002]		
GAD	−0.004	−0.908	0.005	0.368			

*GAD, General anxiety disorder scores*.

## Discussion

While the consolidation of the DMN into a unified network marks a developmental achievement critical for proper brain functioning [for a systematic review and meta-analysis see Mak et al. (85)], very little research examined DMN connectivity across childhood and adolescence, with nearly none testing its maturation in relation to stress-related symptoms and markers of risk and resilience. In the current study, we examined the association between genetic variability on the OT system and DMN connectivity at the transition to adolescence. Our goal was to expand knowledge on how ELS and psychological manifestations of stress undermine the DMN's ability to function as a coherent network whereas better innate functionality of the OT system may facilitates DMN maturation. Utilizing a prospective longitudinal study and following children across the first decade of life, we found that exposure to ELS, cumulative genetic risk on oxytocin-pathway genes, avoidant behavioral response to trauma reminders in early childhood, and the diagnosis of an anxiety disorder in late childhood cumulatively explained 25% of the variance in DMN connectivity in preadolescence. Overall, our findings point in four important directions. First, greater inborn functionality of the OT system sustains maturation of the DMN and may serve to buffer the effects of chronic early stress on DMN connectivity. Second, young children's avoidant response to trauma reminders in the context of chronic trauma serves as an important early indicator of difficulties in neural maturation a decade later. Third, the existence of a well-defined anxiety disorder bears negative consequences for the development of the DMN, a core system sustaining the emerging self. Finally, independent of ELS exposure, these factors cumulatively predict DMN connectivity in preadolescence and when appearing together across childhood may chart a risk trajectory for the maturation of the DMN.

Our findings are the first to demonstrate how the interplay of genetic factors and stress-related symptoms as they mutually influence each other over time shapes DMN connectivity at the transition to adolescence. The DMN is known to be involved in processes implicated in stress regulation, such as hypervigilance and alertness, alterations of intrinsic and extrinsic attention, self-referential mental activity, and recollection of personal experiences (1, 86). Studies in adults have shown that following stress, DMN activation is both reduced (87) and less integrated (88), and various ELS conditions, such as a history of poverty reduces DMN connectivity (88). Much further longitudinal research is thus required to tease various forms of ELS and to chart specific developmental trajectories for each condition that may impair the functioning of core neural networks.

DMN connectivity has been repeatedly associated with PTSD symptoms and anxiety disorders in adults. PTSD has been linked with reduced connectivity of the DMN (13, 14, 89) and a history of chronic early trauma has been associated with reduced DMN connectivity in adulthood, with the degree of impairment increasing as a function of the severity of dissociative symptoms (89). Furthermore, mindfulness-based psychotherapy helped increase DMN connectivity in veterans suffering from war-related PTSD and the improvement correlated specifically with reduction in avoidant symptoms (13), consistent with our findings on the specific associations between DMN connectivity and avoidant symptoms. Similarly, PTSD patients exhibited shorter activations of the DMN after exposure to trauma reminders that correlated with the severity of symptoms (90). Trauma-focused cognitive behavior therapy rescued DMN activity duration of activation and brought it back to normality and other stress-related psychopathologies linked with alterations in DMN activation patterns in adults (91, 92). Our findings highlight avoidant response to trauma reminders in early childhood, which is associated with PTSD chronicity in childhood (47), as an early marker of disrupted neural maturation and underscores the need to identify young children with avoidant symptoms and avoidant behavior in contexts of chronic stress and trauma as those who require the most rapid and targeted interventions.

In addition to PTSD, anxiety and stress-related disorders in adults have been linked with disruptions to DMN functionality (91, 92) and our findings are the first to show similar associations in children. Comorbidity between depression and anxiety was associated with reduced connectivity in the anterior regions of the DMN and increased connectivity in the posterior regions. During task, when DMN activity is typically suppressed, psychosomatic patients with anxiety disorders showed reduced activation in selected DMN nodes (92). In adults, disruptions to DMN alpha connectivity were related to anxious and depressive symptoms (29). The current findings indicate that when an anxiety disorder is consolidated by late childhood, this poses a risk for the development of DMN connectivity during the 10–13-years-old period described by Sherman et al. (19) as a sensitive window for the maturation of the DMN.

The current study expands our previous knowledge by linking genetic variability on the extended OT system with functionality of the DMN in children in the context of chronic early life stress. Based on the literature linking OT to stress-related psychopathologies, such as PTSD and anxiety (70), the OT system has been considered as a potential route for prevention and intervention in stress-related disorders (93). Our findings show that cumulative genetic risk on the extended OT system and avoidant symptoms in early childhood were each uniquely predictive of disruptions to DMN connectivity in early adolescence, above and beyond stress exposure, adding to the discussion on the role of the OT system in brain maturation in the context of chronic early stress. These findings suggest that there may be non-circumstantial factors that pose a risk to functionality of the DMN. Even among children reared in low-risk contexts, those born with more risky dispositions and develop avoidant responses that mature into a full-blown anxiety disorder can suffer a protracted maturation of the DMN. We have previously shown that in these children greater salivary oxytocin was associated with better immune biomarkers and both oxytocin and immune-system functionality buffered the effect of trauma on the development of anxiety disorders (94). This suggests that multiple indices of OT-system functionality may serve as indices of resilience and buffer the effects of stress on outcome in children exposed to chronic stress, trauma, and adversity.

Our findings have several clinical implications for children growing up amidst chronically stressed conditions. First, boosting the child's OT system, whether through intranasal administration or via bolstering parent-child synchrony, parental touch, and physical contact which have shown to increase OT production (95, 96), may provide a buffer against stress-related psychopathologies. Thus, early interventions aimed at increasing child-parent synchronization and physical touch or nasally administrating OT may serve as protective factors for brain development. Second, interventions focusing on reducing avoidant behavior in response to ELS may protect children from the long-lasting effects of ELS on the DMN. Indeed, studies show that therapeutic strategies, whether cognitive or emotional focusing on reducing avoidant behavior are highly successful in reducing the severity of GAD in adults (97) and our findings suggest that the same mechanisms may operate in children.

Several study limitations should be taken into consideration in the interpretation of the findings. First, consistent with much prior research we treated the DMN as a unitary system; however, several recent studies have demonstrated that the DMN comprises several interrelated subsystems (1, 98–100). Similarly, we treated the cumulative genetic risk system as a unitary factor in which each gene received equal weight and it is possible that one or more genes are more important in the current context. Future research should address this possibility by statistically determining the importance of each gene across subjects toward the computation of a more nuanced cumulative variable. In addition, we did not target other neural systems associated with the DMN or OT. Further, there are external factors that predict DMN connectivity in children that were not included in current study. For example, maternal depression, anxiety, and post-traumatic symptoms, which we previously found to be linked with DMN maturation (29). Thus, in order to draw a more comprehensive picture, future research should take into account, in addition to child's characteristics, also the child's surrounding envelope. Finally, some of the findings are only marginally significant, such as the difference in DMN connectivity between children high and low in avoidant behavior and children high and low in anxiety disorder, and these findings should be treated with caution. Nevertheless, it is important to note that while these variables were not significant when tested independently, taken together, they had a significant cumulative effect and when appearing on top of each other across development were found to impair the development of the DMN.

Despite the above limitations, the current study expends previous knowledge by demonstrating the long-lasting effect of ELS on the developing brain. This is the first study to longitudinally test the cumulative impact of genetic, behavioral, and clinical factors as they unfold across development on maturation of a core neural system. Our findings highlight the role of the oxytocin system for brain maturation during a sensitive period of the transition to adolescence and may have important implication for both research on the developing brain and the construction of targeted interventions for children exposed to chronic stress, war, and trauma.

## Data Availability Statement

The datasets analyzed in this article are not publicly available. Request to access the datasets should be directed to feldman.ruth@gmail.com.

## Ethics Statement

The studies involving human participants were reviewed and approved by Ethics Committee of Bar Ilan University. Written informed consent to participate in this study was provided by the participants' legal guardian/next of kin.

## Author Contributions

RF designed the longitudinal study and wrote the paper. JL designed and conducted the MEG experiment. MZ-W analyzed the MEG data and wrote the paper. RE conducted the genetic analysis.

## Conflict of Interest

The authors declare that the research was conducted in the absence of any commercial or financial relationships that could be construed as a potential conflict of interest.
